# Downregulation of *TTF1* in the rat hypothalamic ARC or AVPV nucleus inhibits *Kiss1* and *GnRH* expression, leading to puberty delay

**DOI:** 10.1186/s12958-021-00710-7

**Published:** 2021-02-23

**Authors:** Shaolian Zang, Xiaoqin Yin, Pin Li

**Affiliations:** Department of Endocrinology, Shanghai Children’s Hospital, Shanghai Jiao Tong University, Shanghai, 200062 People’s Republic of China

**Keywords:** Thyroid-specific transcription factor 1 (*TTF1)*, *Kiss1*, Gonadotropin-releasing hormone (*GnRH)*, RNA interference (RNAi), Stereotaxic injection, Puberty

## Abstract

**Background:**

*TTF1* is a transcription factor that is expressed in the hypothalamus after birth and plays crucial roles in pubertal development. *TTF1* may regulate the expression of the *Kiss1* gene, which may drive puberty onset in the hypothalamic arcuate (ARC) and anterior ventral paraventricular (AVPV) nuclei.

**Methods:**

A dual-luciferase reporter assay was used to detect binding between *TTF1* and the *Kiss1* gene promoter. To investigate the effects of *TTF1*, we modified *TTF1* expression in cell lines and in the ARC or AVPV nucleus of 21-day-old female rats via lentivirus infection. *TTF1* and other puberty onset-related genes were detected by qRT-PCR and western blot analyses.

**Results:**

The in vitro data indicated that *TTF1* knockdown (KD) significantly reduced *Kiss1* and *GnRH* expression. Overexpression (OE) of *TTF1* promoted *Kiss1* expression. In vivo, the expression of *Kiss1* and *GnRH* decreased significantly in the rats with hypothalamic ARC- or AVPV-specific TTF1 KD. The TTF1-KD rats showed vaginal opening delay. H&E staining revealed that the corpus luteum was obviously reduced at the early puberty and adult stages in the rats with ARC- or AVPV-specific TTF1 KD.

**Conclusion:**

*TTF1* bound to the promoter of the *Kiss1* gene and enhanced its expression. For 21-day-old female rats, decreased *TTF1* in the hypothalamic ARC or AVPV nucleus resulted in delayed vaginal opening and ovarian abnormalities. These observations suggested that *TTF1* regulates puberty onset by promoting the expression of *Kiss1* and plays an important role in gonad development.

## Background

In mammals, the maturation of reproductive function begins with the onset of puberty, a process regulated by the hypothalamic-pituitary-gonadal (HPG) axis and tightly coordinated by a complex network of excitatory and inhibitory genetic factors. The HPG axis is first activated during the embryonic phase and early postnatal days, and it is subsequently suppressed in childhood. However, the HPG axis is reactivated, and its activation culminates at the onset of puberty [1]. At puberty initiation, the secretion of gonadotropin-releasing hormone (GnRH), which acts on the gonads, increases significantly in the hypothalamus, causing a surge in sex steroid hormones and leading to gonadal development [2]. Genetic and/or environmental factors affect the pubertal development time of mammals. Using epidemiological information, previous researchers determined that 50–80% of abnormal adolescent development time was associated with genetic factors [3]. However, the regulatory mechanism of GnRH neurons is very complicated, and the specific molecular mechanism is not fully understood. It is currently agreed that Kisspeptin and its receptor G protein-coupled receptor 54 (GPR54) directly regulate the release of GnRH. Kisspeptin is a key upstream regulator of GnRH and pubertal development [4–6]. The arcuate (ARC) and anterior ventral paraventricular (AVPV) nuclei are well known for their abundant expression of the *Kiss1* gene [7]. Large numbers of studies have suggested that these two regions have different effects on reproduction. The ARC is negatively regulated by oestrogen. It is responsible for the pulsed release of luteinizing hormone (LH) to form the reproductive cycle, while the AVPV is positively regulated by oestrogen and is responsible for the generation of the preovulatory LH surge that stimulates ovulation [8, 9]. Consistent with these studies, oestradiol-mediated positive feedback increases the excitability of these neurons and glutamate transmission to AVPV neurons [10]. In fact, oestrogen receptor α (ERα) expression on *Kiss1*-positive neurons is critical for the positive or negative feedback of oestradiol because Kisspeptin-specific ERα knockout (KERKO) mice were shown to exhibit high-frequency LH pulses and did not exhibit an oestradiol-induced LH surge [9]. Moreover, the expression of Kisspeptin in the region around the third ventricle (3 V) is sex biased, and the number of *Kiss1*-expressing neurons in females is much greater than that in males. Researchers hypothesized that this was necessary for the LH surge before ovulation [11–13].

Thyroid-specific transcription factor-1 (TTF-1), also known as Nkx2–1, thyroid-specific enhancer-binding protein (T/EBP) or TITF1, is located on chromosome 14q13.3 and contains three exons. The TTF1 protein, which consists of 371 amino acids, is a 38-kDa nuclear DNA-binding protein [14, 15]. Previous studies found that the amino acid sequence of TTF1 has 98% similarity among the human, rat and mouse genomes and that the 60-amino acid homology domain is highly conserved [16]. *TTF1* is mainly expressed in the forebrain, pituitary gland, lung and thyroid [17]. In recent years, studies have shown that *TTF1* may be an upstream regulator of the *Kiss1* gene [18]. Researchers have found that the ventromedial nucleus and dorsal nucleus of the hypothalamus did not develop in mice carrying a *TTF1* gene null mutation, causing the wall of the third ventricle to fuse on its ventral side and resulting in the absence of the ARC [17]. Correa et al. [19] found that conditional knockout of *TTF1* in the ventromedial hypothalamus interfered with the normal development of neurons in the central nervous system. *TTF1* is critical for functional hypothalamic and pituitary morphology in mammals. Moreover, researchers found that when the secretion of sex hormones was at the basal level, the level of *TTF1* mRNA in the hypothalamus increased during development [20]. Kim et al. [20] found that the expression of *TTF1* reached a significant peak before puberty onset between postnatal days 26 and 27. These results demonstrated that *TTF1* is a central component of the puberty process that might be involved in the central activation of mammalian adolescence.

The purpose of this study was to investigate the probable effects of *TTF1*. Our study aimed to clarify the potential mechanism and phenotype resulting from *TTF1* gene downregulation in the rat hypothalamus and ND7–23 neuron cell lines or *TTF1* gene upregulation in GT1–7 cells. This research will help us understand the initial process of puberty.

## Materials and methods

### Animals

Twenty-one-day-old female Sprague-Dawley (SD) rats (body weight, 50–60 g) (*n* = 114) were purchased from Shanghai SLAC Laboratory Animal Co. (Shanghai, China) and randomly separated into four groups: injection of lentivirus-enhanced green fluorescent protein (LV-EGFP) in the ARC, injection of LV-EGFP in the AVPV, injection of LV-TTF1-shRNA in the ARC and injection of LV-TTF1-shRNA in the AVPV. The rats were subjected to bilateral microinjections between 12:00 and 18:00. Then, they were housed five per cage under controlled temperature (21 ± 2 °C) and humidity (55 ± 10%) conditions with a 12 h light/12 h dark cycle (lights on between hours 7:00–19:00). Food and water were available ad libitum. We inspected the vaginal openings of the rats every morning between 9:00 and 9:30 AM daily from postnatal day 28 (PND28). Finally, the rats were euthanized at the juvenile (PND25), early puberty (PND35), and adult stages (PND42) between 9:00 and 17:00. All procedures were approved by the Institutional Animal Care and Use Committee of Shanghai, China (Ethics review number: 2018022).

### Cell culture

ND7–23 is a neuronal cell line that endogenously expresses not only the *TTF1* gene but also the *Kiss1* and *GnRH* genes. It was used to verify the knockdown efficiency of the lentivirus. ND7–23 and 293 T cells were purchased from the Cell Bank of the Chinese Academy of Sciences (Shanghai, China). GT1–7 is a hypothalamic neuronal cell line. The GT1–7 cells used in this study were kindly provided by the Shanghai Clinical Center for Endocrine and Metabolic Diseases, Shanghai Jiaotong University. The cells were maintained in Dulbecco’s modified Eagle’s medium (DMEM, Gibco, NY, USA) supplemented with 10% foetal bovine serum (FBS, Gibco), 100 U/mL penicillin and 100 μg/mL streptomycin. The cells were cultured in a humidified atmosphere containing 5% CO_2_ at 37 °C.

### *TTF1* shRNA construction

**Fig. 1:**
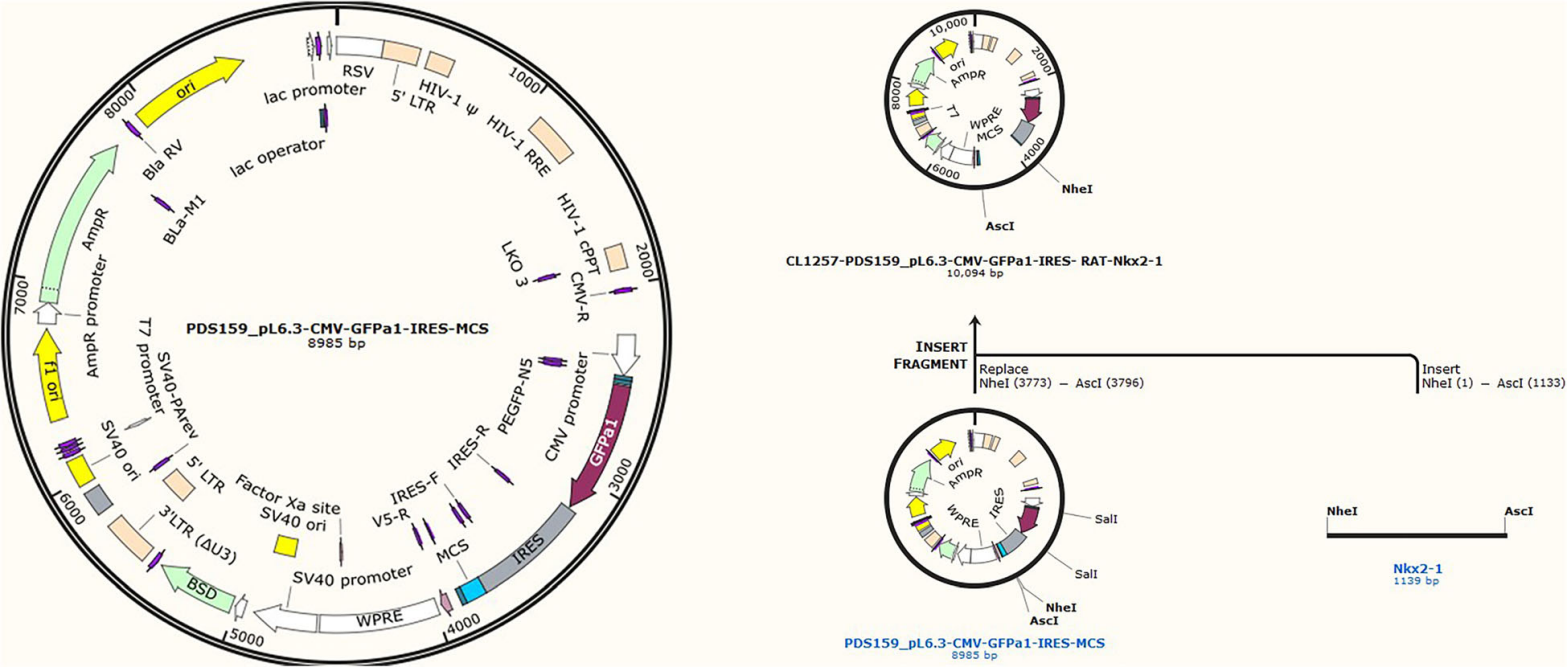
Lentivirus vector profile

All oligo sequences were designed with an online design tool provided by Invitrogen (http://www.invitrogen.com/rnai). The oligo sequences of the rat *TTF1* shRNA are shown in Table 1. Additionally, we constructed a TTF1 overexpression plasmid (NM_013093.1). A restriction enzyme site and a Kozak sequence (GCCACC) were added to the 5′ end of the sequence. The oligonucleotides were used to construct plasmid vectors. A schematic diagram of the vector construction is shown in Fig. 1. After correct identification, the recombinant plasmid was amplified in large amounts. Fluorescence immunochemistry was used to determine the transfection efficiency. qRT-PCR and western blotting were applied to detect the interference efficiency of TTF1-shRNA.

**Table 1:** Sequences of the shRNA oligos targeting rat TTF1 and the negative control oligos

Name	Sequences(5′-3′)
TTF1-shRNA-F	CACCGGAGGAAAGCTACAAGAAAGTCGAAACTTTCTTGTAGCTTTCCTCC
TTF1-shRNA-R	AAAAGGAGGAAAGCTACAAGAAAGTTTCGACTTTCTTGTAGCTTTCCTCC
Negative control	CTAAGGTTAAGTCGCCCTCGC

### Lentivirus infection

The ability of the TTF1-shRNA constructs to reduce TTF1 levels was measured and compared to that of the negative control lentivirus (LV-NC) in the ND7–23 cell line. The cells were plated in 6-well plates at 2 × 10^5^ cells per well in 10% FBS. Twenty-four hours later, the cells were transduced with the viruses. Seventy-two hours after transfection, total RNA and protein were extracted from the ND7–23 cells to detect the interference efficiency of the TTF1-shRNAs. Additionally, we used the above methods to transfect GT1–7 cells with the *TTF1* overexpression plasmids to explore the regulatory relationship between *TTF1* and *Kiss1*.

### Bilateral microinjection

As described in a previous study [21, 22], surgery was performed at PND21 to allow 72 h of infection for maximum expression of TTF1-shRNA before puberty onset. Twenty-one-day-old female rats were positioned in a stereotaxic instrument after they were deeply anaesthetized with 1% sodium pentobarbital (0.5 mL/100 g body weight). The skin and periosteum were incised to expose the bregma point. At the beginning of the experiment, 0.5 μL of Brilliant Blue was injected into the ARC or AVPV nucleus to visualize the position of the nuclei in each animal. Two microlitres of TTF1-shRNA or LV-EGFP lentivirus (1.8 × 10^9^ transducing units [TU]/mL) was microinjected bilaterally into the ARC and AVPV nuclei using a 10-μL syringe (Gauge, China) with a glass needle connected to its tip. After injection, we left the needle in place for 5–10 min and then removed it slowly. The coordinates for the ARC (0.4 mm lateral, 1.6 mm posterior to bregma, 9.4 mm below the surface of the dura) and AVPV (0.2 mm lateral, 1.2 mm anterior to bregma, 8.0 mm below the surface of the dura) were determined from the Rat Brain Atlas (Paxinos and Watson, Fifth Edition) and were used previously [23, 24].

### Tissue preparation

Rats were weighed and delivered a fatal dose of 3% sodium pentobarbital (Sigma, USA) to induce euthanasia. The anogenital distance (AGD) of the rats was measured with a Vernier calliper. The rats were decapitated to collect the whole brain, which was subsequently frozen on dry ice. The hypothalamic tissues containing the AVPV and ARC nuclei were separated and immediately placed into liquid nitrogen. The samples were stored at − 80 °C and used for qRT-PCR (TaKaRa, Japan) and western blotting. The uterine and ovarian tissues were collected and weighed to calculate the uterine and ovarian organ coefficients. For immunofluorescence localization, we perfused saline with 10% formalin through the hearts of rats. Intact brains were removed from the skulls and stored in 10% formalin overnight at 4 °C. The brains were transferred into 20 and 30% sucrose when they were totally settled. Serial 20-μm coronal sections containing the AVPV or ARC were obtained using a freezing microtome (Thermo, MI, USA).

### Quantitative real-time PCR (qRT-PCR)

Total RNA was extracted from the tissues (ARC or AVPV) and cultured cells using TRIzol Reagent (Invitrogen, CA, USA) following the manufacturer’s instructions. The RNA concentrations were determined by spectrophotometric traces (Nanodrop, Thermo, Wilmington, DE). Total RNA (1 μg) was transcribed into cDNA in a volume of 20 μL using 5X PrimeScript RT Master Mix (TaKaRa, Japan). We measured the mRNAs of interest using the SYBR Premix Ex Taq qPCR system (TaKaRa, Japan). The primers (Table 2) were synthesized by Shanghai Sangon Biotech Co., Ltd., and β-actin was used as an internal reference. All PCRs were carried out using a Roche Real-Time PCR System in a total reaction volume of 10 μL containing 3 μL of cDNA (50–100 ng/μL), 2 μL of primers (1 μL of each primer), and 5 μL of SYBR Premix Ex Taq. The PCR conditions were as follows: initial denaturation and enzyme activation at 95 °C for 600 s, followed by 45 cycles of denaturation at 95 °C for 10 s, annealing at 60 °C for 30 s, and extension at 72 °C for 1 min. All samples were run in triplicate for each gene. The relative expression of genes was determined using the 2^-ΔΔCt^ method with normalization to β-actin expression.

**Table 2:** Primers used for qRT-PCR

Gene	Forward primers(5′-3′)	Reverse primers(5′-3′)
*Kiss1* (Rat)	AGCTGCTGCTTCTCCTCTGT	AGGCTTGCTCTCTGCATACC
*TTF1* (Rat)	GGACGTGAGCAAGAACATGG	GCCGACAGGTACTTCTGCTG
*GnRH* (Rat)	CCGCTGTTGTTCTGTTGACTGTG	GGGGTTCTGCCATTTGATCCTC
*β-actin* (Rat)	TGCCGCATCCTCTTCCT	GGTCTTTACGGATGTCAACG
*TTF1* (mouse)	GGGCCAGGTCTCTAGCCTATC	CTCACCAGGTCCGACCATAAA
*Kiss1* (mouse)	CTCTGTGTCGCCACCTATGG	AGGCTTGCTCTCTGCATACC
*GnRH* (mouse)	TGATCCTCAAACTGATGGCCG	CGCAACCCATAGGACCAGTG
*β-actin* (mouse)	AAGATCAAGATCATTGCTCCTCC	GACTCATCGTACTCCTGCTTGC

### Western blotting

Samples were homogenized in M-PER® Mammalian Protein Extraction Reagent lysis buffer (Thermo, MI, USA) containing protease inhibitor cocktail and 0.5 M EDTA (Thermo, MI, USA) (1:100 dilution). The supernatant was collected after centrifugation at 12000 rpm for 30 min at 4 °C. The protein concentration was determined using the BCA Protein Assay Kit (Thermo, MI, USA). The protein samples were separated using sodium dodecyl sulfate-polyacrylamide gel electrophoresis (SDS-PAGE) and then transferred onto polyvinylidene fluoride (PVDF) membranes (Millipore, MA, USA). The membranes were blocked with 5% nonfat milk at room temperature for 1 h and then incubated with anti-TTF1 (1:500, Abcam, MA, USA), anti-Kiss1 (1:400, Abcam, MA, USA) or anti-β-tubulin antibody (1:1000, CST, MA, USA) at 4 °C overnight. Subsequently, the membranes were rinsed three times with Tris-buffered saline with 0.1% Tween 20 (TBST) every 10 min and probed with an HRP-labelled secondary antibody (1:10000, Jackson, PA, USA) at room temperature for 1 h. After three additional rinses with TBST, the membranes were visualized using the ECL system. The grey values of the protein bands were analysed using ImageJ software.

### Haematoxylin-eosin (H&E) staining

Rat ovaries were collected and fixed in 10% neutral-buffered formalin. Subsequently, they were dehydrated in a series of ethanol concentrations, cleared in xylene, blocked in paraffin wax, and cut into serial 4 μm sections. Six representative sections were selected from each ovary; these were deparaffinized in xylene, hydrated in a series of ethanol concentrations, and stained with H&E. Finally, the sections were analysed under an optical microscope.

### Dual-luciferase reporter assay

The JASPAR database was used to identify the binding site between *TTF1* and the *Kiss1* promoter. A *Kiss1*-promoter luciferase plasmid and an empty plasmid (pGL4.10) were constructed. Two hundred ninety-three T cells were seeded at a cell density of 2 × 10^5^/cells mL. They were divided into four groups: the *Kiss1*-promoter + NC group, the *Kiss1*-promoter + *TTF1*-OE group, the pGL4.10 + NC group, and the pGL4.10 + *TTF1*-OE group. Lipofectamine 2000 (Invitrogen, CA, USA) was used to transfect the *Kiss1* promoter plasmid and the Renilla luciferase-containing plasmid. The activity of firefly luciferase relative to that of Renilla luciferase was determined using the Dual-Luciferase Reporter Assay Kit (Vazyme, China).

### Statistics

All statistical analyses were performed using SPSS software (version 13; SPSS, Inc., Chicago IL, USA). The data are presented as the mean ± S.E.M. The data were first subjected to normality and equal variance tests. The data that passed these two tests were then analysed by Student’s t-test to compare two groups using SPSS. The intergroup differences among multiple groups were analysed using one-way analysis of variance (ANOVA). Then, we plotted the data with GraphPad Prism 8.0 software (GraphPad, San Diego, CA, USA). *P* < 0.05 was regarded as statistically significant.

## Results

*TTF1* knockdown inhibited the expression of *Kiss1* and *GnRH* in ND7–23 cells.

**Fig. 2:**
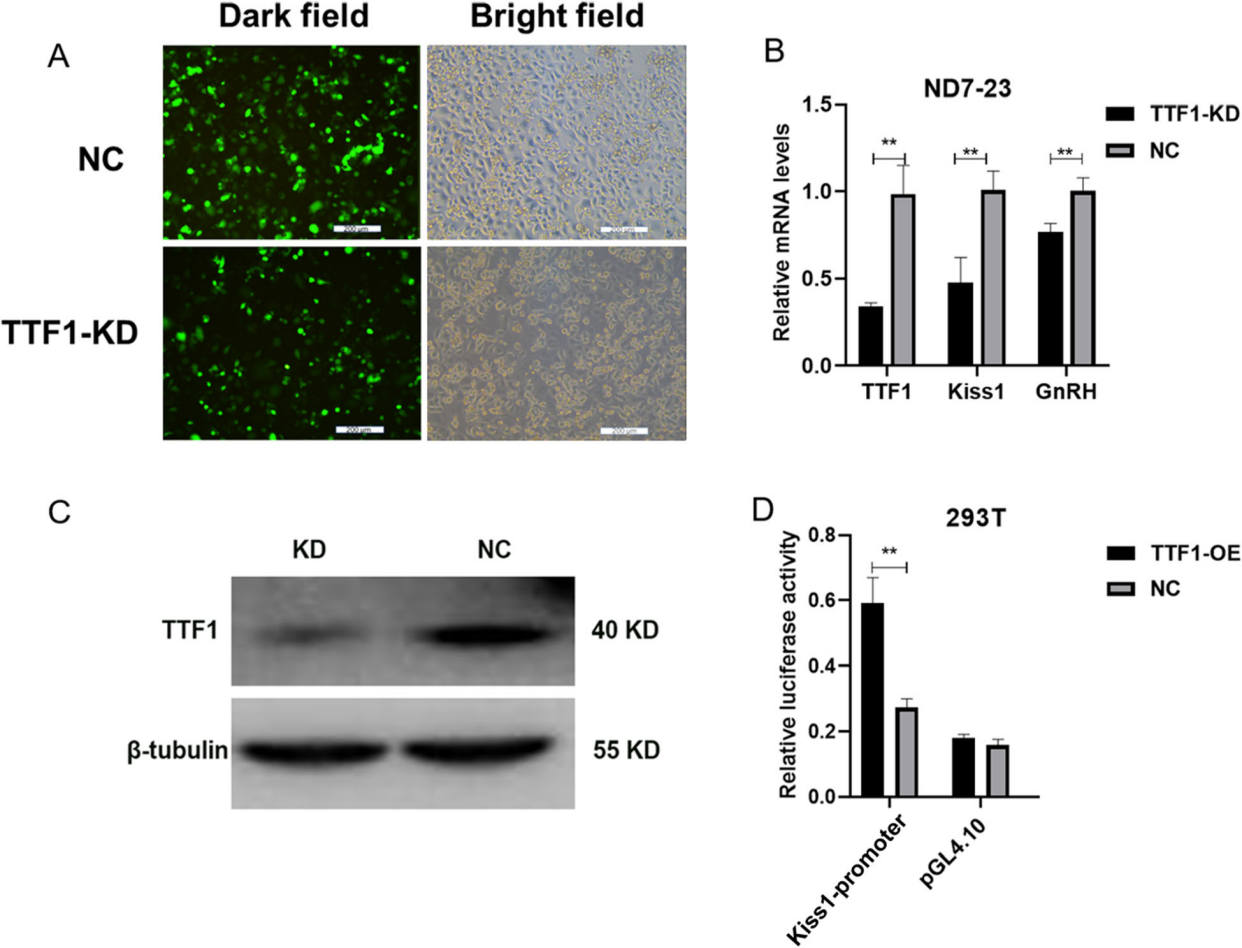
*TTF1* gene knockdown in ND7–23 cells downregulated the expression of *Kiss1* and *GnRH* and decreased *TTF1* binding to the *Kiss1* promoter. **a** ND7–23 cells were infected with LV-TTF1-shRNA for 72 h. EGFP (green) was measured with immunofluorescence microscopy. Optical microscopy was used to obtain bright field images. **b** *TTF1*, *Kiss1* and *GnRH* mRNA levels were analysed with qRT-PCR after transfection with LV-TTF1-shRNA. Cells transfected with LV-EGFP were used as a negative control. **c** TTF1 protein concentration was determined by western blotting analysis. **d** The effect of *TTF1* on the activity of the *Kiss1* promoter was detected with a Dual-Luciferase Reporter Assay. TTF1-KD, TTF1-knockdown group. TTF1-OE, TTF1-overexpression group. NC, lentivirus negative control group. Scale bars, 200 μm **a**. The results are shown as the mean ± S.E.M. **P < 0.01

We successfully designed RNA interference sequences for the rat *TTF1* gene and inserted them into a lentiviral vector. After infection for 72 h, enhanced green fluorescent protein (EGFP) was expressed in ND7–23 cells (Fig. 2a). qRT-PCR and western blot analysis showed that LV-TTF1-shRNA transfection significantly decreased the expression of the *TTF1* gene (Fig. 2b and c). *TTF1* knockdown reduced the mRNA levels of *Kiss1* and *GnRH* (Fig. 2b). A dual-luciferase reporter assay demonstrated that *TTF1* could bind to the promoter of the *Kiss1* gene (Fig. 2d). These findings indicated that *TTF1* could bind to the *Kiss1* promoter and activate the transcription of the *Kiss1* gene.

### Overexpression of *TTF1* promoted the expression of *Kiss1* in GT1–7 cells

**Fig. 3:**
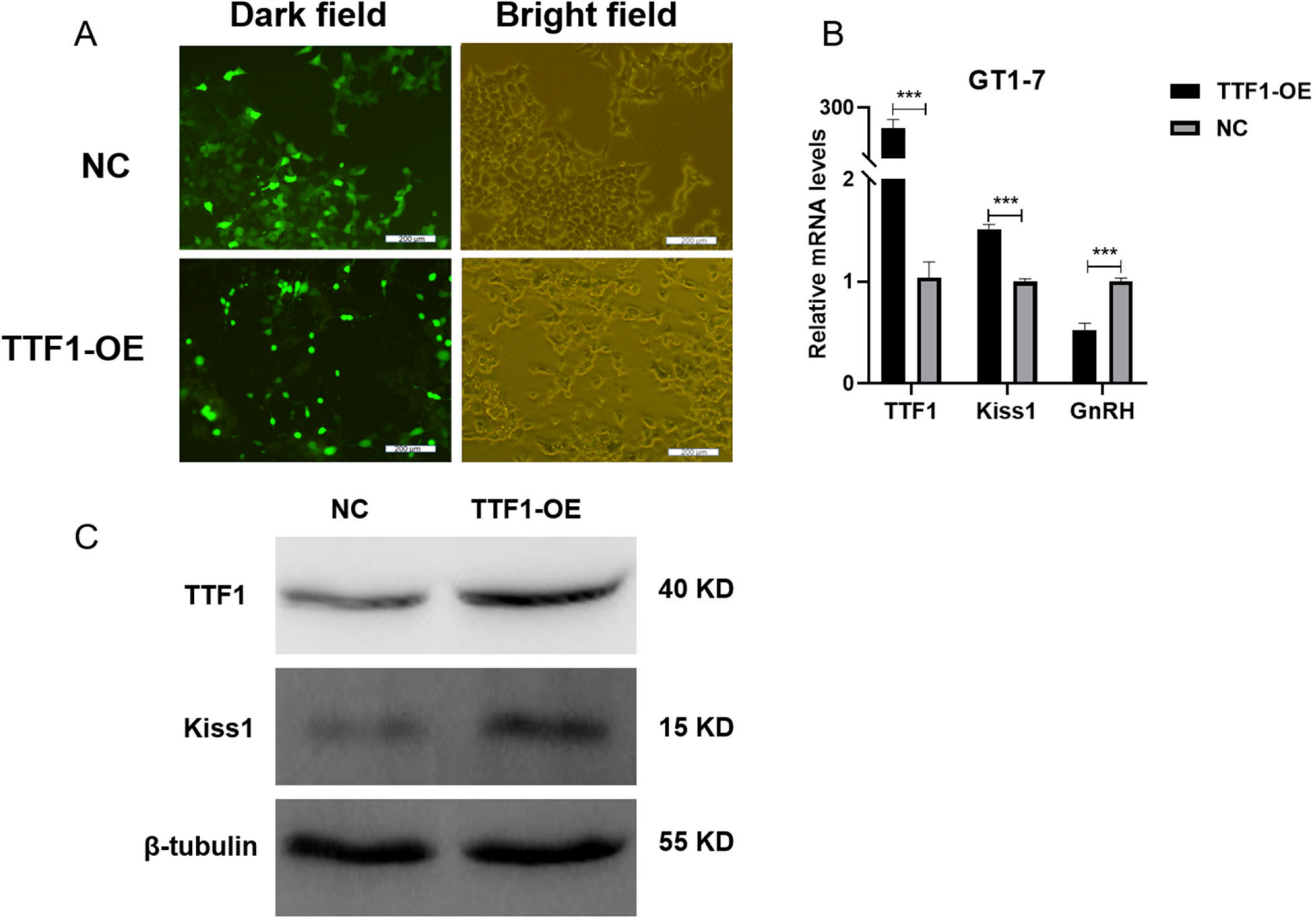
*TTF1* gene overexpression in GT1–7 cells promoted the expression of *Kiss1*. GT1–7 cells were infected with *TTF1* overexpression plasmids for 72 h. **a** EGFP (green) was measured with immunofluorescence microscopy. Optical microscopy was used to obtain bright field images. **b** *TTF1*, *Kiss1* and *GnRH* mRNA levels were analysed with qRT-PCR after transfection. Cells transfected with LV-EGFP were used as a negative control. **c** TTF1 and Kiss1 protein concentrations were determined by western blotting analysis. TTF1-OE, TTF1-overexpression group. NC, lentivirus negative control group. Scale bars, 200 μm **a**. The results are shown as the mean ± S.E.M. ***P < 0.001

We transfected GT1–7 cells with a *TTF1* overexpression plasmid to explore the regulatory relationship between *TTF1* and *Kiss1*. After infection for 72 h, EGFP was expressed in GT1–7 cells (Fig. 3a). qRT-PCR showed that transfection of the *TTF1* overexpression plasmid significantly increased the expression of the *TTF1* and *Kiss1* genes (Fig. 3b and c). However, the expression of *GnRH* mRNA decreased (Fig. 3b). These results suggested that *TTF1* promoted *Kiss1* expression in GT1–7 cells.

### Anatomical localization of EGFP in the ARC and AVPV

**Fig. 4:**
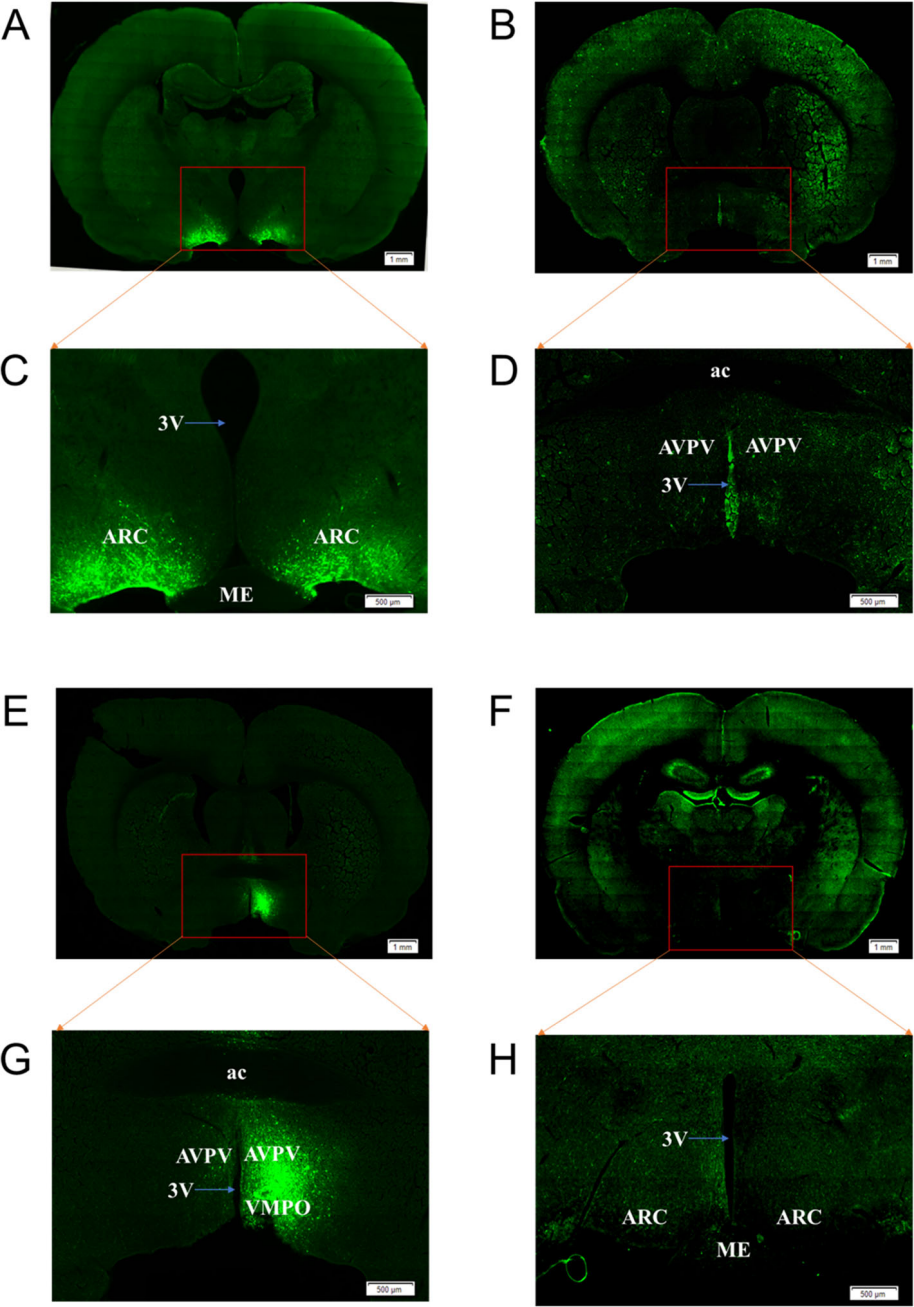
Localization of GFP expression in the rat ARC and AVPV. Neuroendocrine cells are shown by GFP-staining (green). Representative images showing GFP distribution in the ARC **a** and **c** and AVPV **b** and **d** after intra-ARC administration are shown. Representative images showing GFP distribution in the AVPV **e** and **g**) and ARC **f** and **h** after intra-AVPV administration are also shown. ARC, Arcuate nucleus. AVPV, Anterior ventral paraventricular nucleus. Three V, the third ventricle. ac, anterior commissure. VMPO, ventromedial preoptic nucleus. ME, Medial eminence. The red arrows represent the corresponding images at high magnification. The blue arrow refers to the 3 V. Scale bars, 1 mm **a**, **b**, **e** and **f**). Scale bars, 500 μm (**c**, **d**, **g** and **f**

LV-TTF1-shRNA was injected into the ARC or AVPV nucleus of 21-day-old female rats via bilateral microinjection, and rats injected with LV-NC were used as a negative control. We referred to the Rat Brain Atlas (Paxinos and Watson, Fifth Edition). We first injected 1 μL of lentivirus into the ARC and AVPV of 21-day-old female rats. After injection, little GFP expression was observed in frozen brain sections. We speculated that the lentivirus did not reach the titre that was reached in the study we referenced [25]. Then, we increased the lentivirus injection volume to 2 μL. EGFP-containing cell bodies and axons were observed at high densities within the ARC (Fig. 4a and c) and AVPV (Fig. 4e and g). Slight expression of EGFP was also observed in the ventromedial preoptic nucleus (VMPO), which has low expression of Kiss1 and GnRH. Critically, for the intra-AVPV and intra-ARC LV-EGFP-injected animals, no EGFP-containing neurons were observed in the corresponding AVPV (Fig. 4b and d) and ARC (Fig. 4f and h), respectively.

### *TTF1* knockdown reduced the expression of *Kiss1* and *GnRH* in female rats

**Fig. 5:**
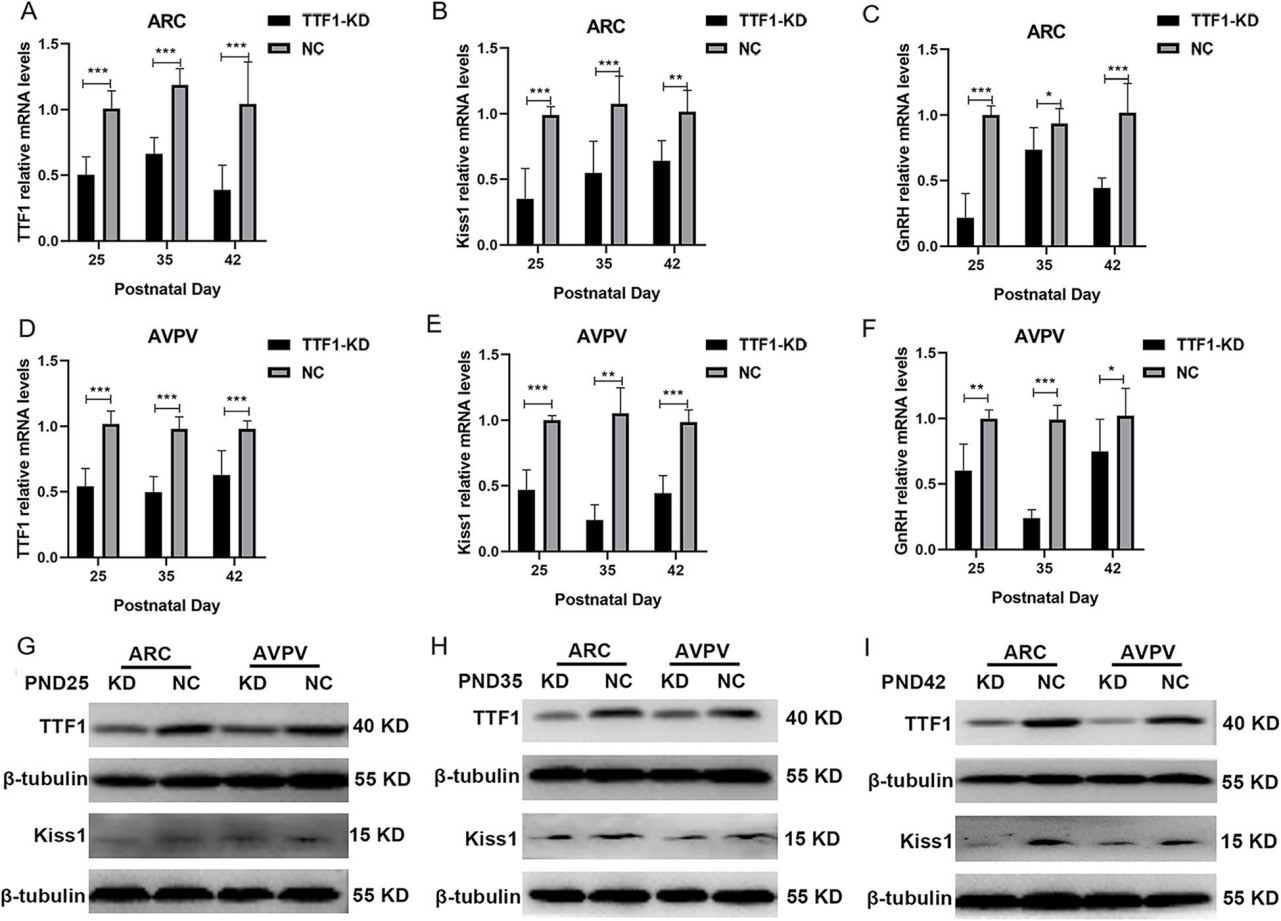
Knockdown of the *TTF1* gene inhibited the expression of *Kiss1* and *GnRH* in both the ARC and AVPV. Twenty-one-day-old female rats were infected with LV-TTF1-shRNA or LV-NC (*n* = 6). **a-c** mRNA levels of *TTF1*, *Kiss1* and *GnRH* in the ARC were detected with qRT-PCR at PND25, PND35 and PND42. **d**-**f** Expression of the *TTF1*, *Kiss1* and *GnRH* genes in the AVPV was detected with qRT-PCR (*n* = 6) at PND25, PND35 and PND42. β-actin was used as the internal reference. **g**-**i** Expression of the TTF1 and Kiss1 proteins in the ARC and AVPV was detected by western blotting at PND25, PND35 and PND42. KD for the TTF1-knockdown group, NC for the lentivirus negative control group. ARC, Arcuate nucleus. AVPV, Anterior ventral periventricular nucleus. The results are shown as the mean ± S.E.M. *P < 0.05, **P < 0.01, ***P < 0.001

To determine whether *TTF1* knockdown in the ARC or AVPV nucleus could influence the expression of *Kiss1* and *GnRH,* as demonstrated in vitro, we injected virus particles into 21-day-old female rats and collected ARC and AVPV tissue at PND25, PND35 and PND42 for mRNA quantitation and protein detection. The expression of the *TTF1* mRNA was reduced significantly in the ARC and AVPV knockdown groups (Fig. 5a and d). TTF1 protein expression decreased significantly in the ARC and AVPV knockdown groups at PND25, PND35 and PND42 (Fig. 5g, h and i). Consistent with the in vitro data, *TTF1* knockdown (TTF1-KD) in the ARC caused a pronounced reduction in *Kiss1* and *GnRH* mRNA abundance (Fig. 5b and c). Downregulation of *TTF1* in the hypothalamic AVPV also reduced *Kiss1* and *GnRH* expression at PND25, PND35, and PND42 (Fig. 5e and f). However, there were no significant changes in Kiss1 protein expression in the juvenile TTF1-KD groups (PND25) (Fig. 5g). We noticed that *Kiss1* expression was reduced in the TTF1-KD groups at the early puberty stage (PND35) and the adult stage (PND42) (Fig. 5h and i). Decreasing *TTF1* expression in the ARC and AVPV during prepuberty selectively reduced *Kiss1* and *GnRH* expression.

### *TTF1* knockdown in the ARC or AVPV nucleus caused delayed puberty in female rats

**Fig. 6:**
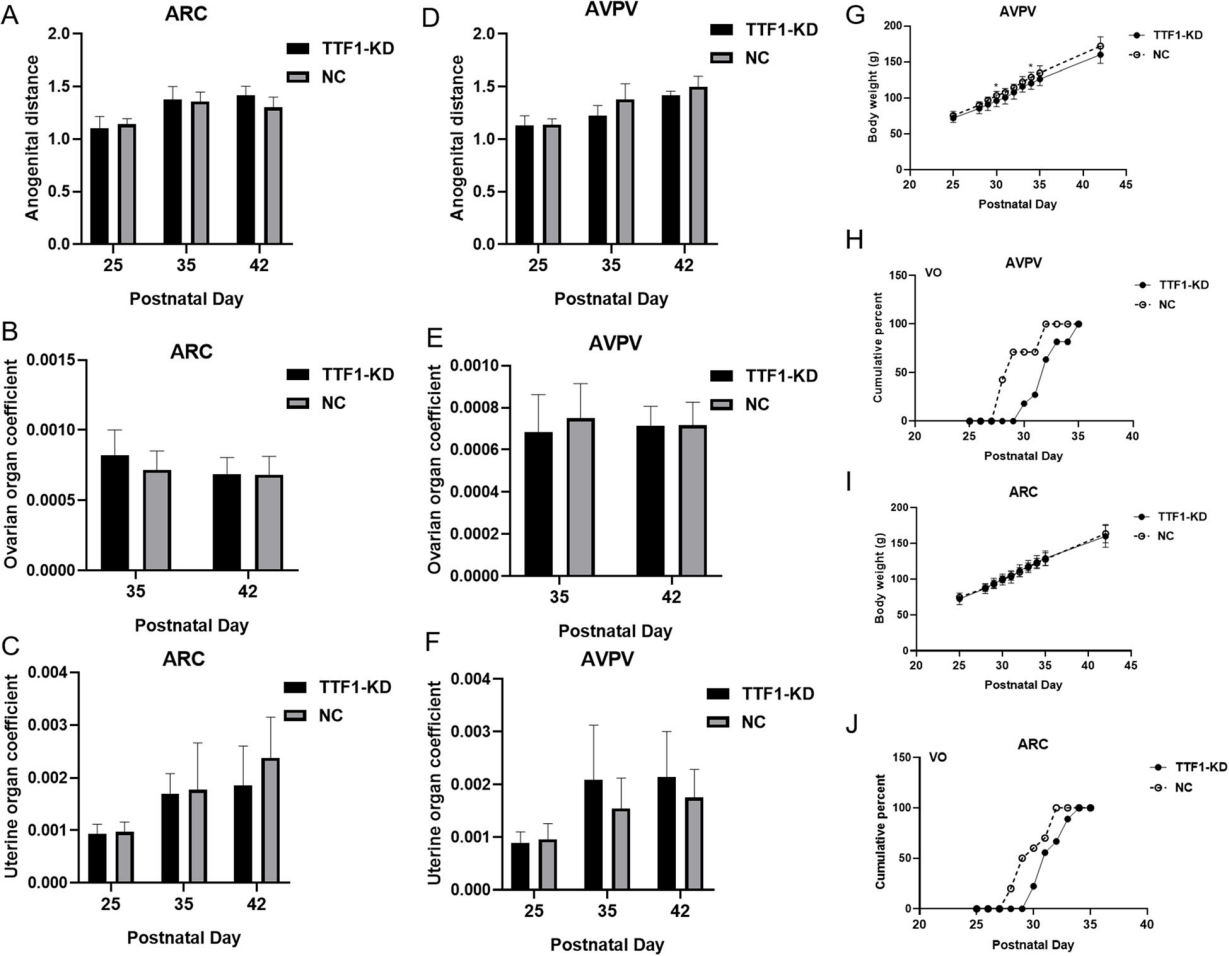
Effects of intra-ARC and intra-AVPV LV-TTF1-shRNA administration on female rat puberty. Twenty-one-day-old female rats were infected with LV-TTF1-shRNA or LV-NC in the ARC or AVPV nucleus. **a-c**) Anogenital distance, ovarian organ coefficient and uterine organ coefficient in the rats injected with the LV-TTF1-shRNA or NC in the ARC were measured at different stages. **d**-**f** Anogenital distance, ovarian organ coefficient and uterine organ coefficient in the rats transfected with the NC or LV-TTF1-shRNA in the AVPV were measured at different stages. **g** and **i**) The body weights of rats receiving virus transfection were measured beginning at PND25. **h** and **j** Cumulative percentages of vaginal openings (VOs) were determined every morning after the intra-AVPV LV-TTF1-shRNA and intra-ARC LV-TTF1-shRNA transfection. Ovarian organ coefficient = ovary weight (g)/body weight (g). Uterine organ coefficient = uterine weight (g)/body weight (g). The data are presented as the mean ± S.E.M. *P < 0.05

TTF1-KD female rats were observed every morning after virus injection to determine the vaginal opening (VO) time. There was no significant difference in anogenital distance, ovarian organ coefficient (ovary weight/body weight), uterine organ coefficient (uterine weight/body weight), or gross body weight (Fig. 6a, b, c, d, e, f and i). However, the rats with TTF1-KD in the AVPV (*n* = 11) showed a significant delay in the age of vaginal opening compared with that of the negative control rats (*n* = 7) (Fig. 6h). The rats with TTF1-KD in the ARC (*n* = 9) also showed a significant VO delay (Fig. 6j). The TTF1-KD groups showed a trend of decreased body weight at the prepuberty stage compared to those of the control groups (Fig. 6g). These data demonstrated that in vivo TTF1 knockdown in the ARC or AVPV nucleus led to delayed initiation of puberty and delayed the normal pubertal process.

### TTF1 knockdown caused abnormal ovary development in female rats

**Fig. 7:**
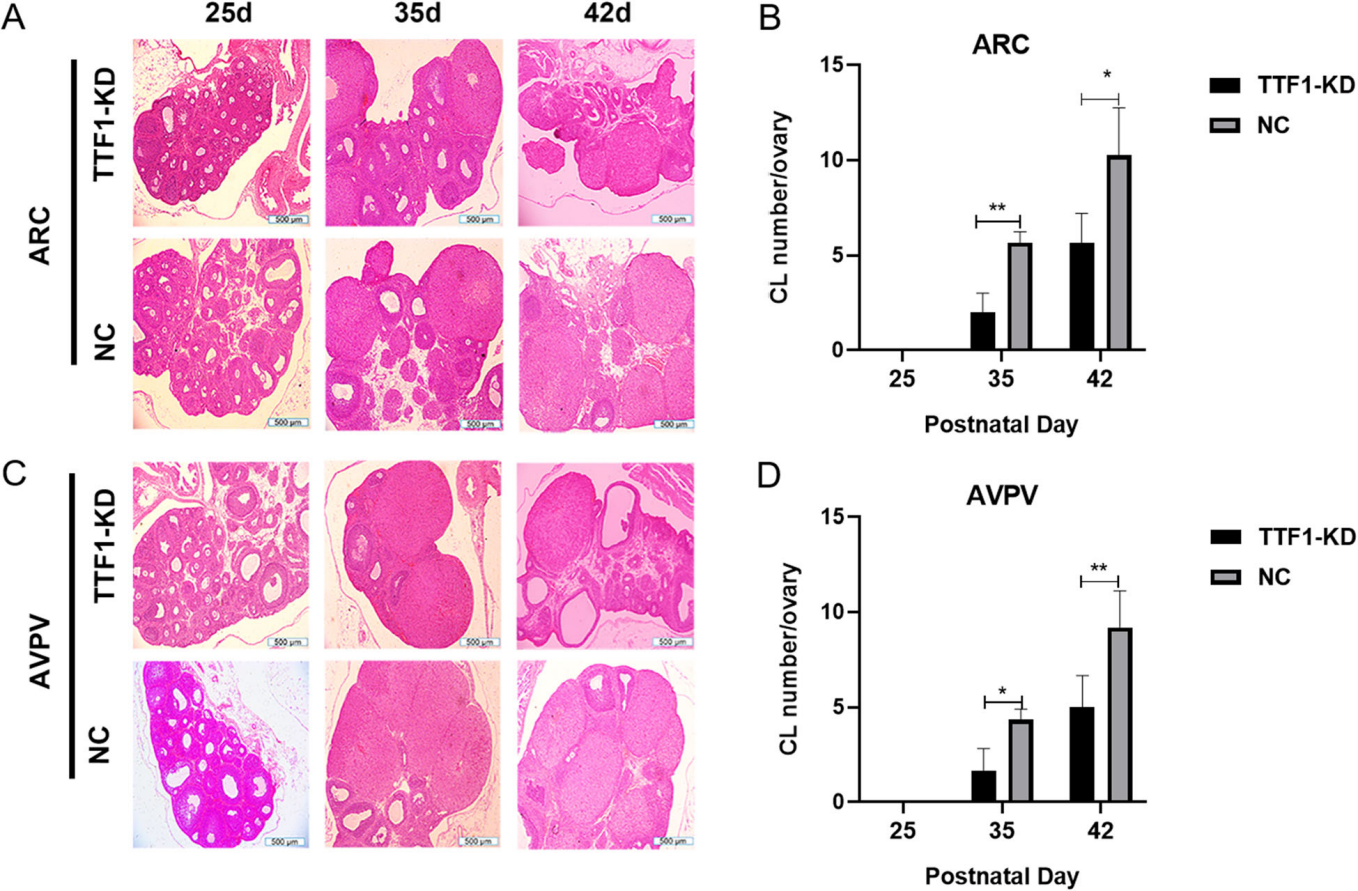
Ovary development phenotypes associated with TTF1 expression. Pathological assessment of follicular development was performed by H&E staining in the ARC-infected group **a** and the AVPV-infected group **c**. **b** The number of corpora lutea (CL) in the ARC-infected group was measured at PND35 and PND42. **d** The number of CL in the AVPV-infected group was detected at PND35 and PND42. Scale bars, 500 μm **a** and **c**. The data are presented as the mean ± S.E.M. *P < 0.05, **P < 0.01

TTF1 regulated the onset of puberty and reproduction. We used H&E staining to observe ovarian morphology and function. At PND35 and PND42, the CL numbers decreased significantly in both the AVPV- and ARC-specific TTF1-KD groups compared with the negative control groups (Fig. 7a-d). These data demonstrated that in vivo silencing of TTF1 in the ARC or AVPV nucleus influenced the development of the ovary and decreased CL numbers.

## Discussion

Pubertal development is a complex process of biological regulation that is affected by many factors. This research aimed to explore whether *TTF1* knockdown in the ARC or AVPV nucleus via bilateral virus microinjection influences rat pubertal development and reproductive function. In in vitro experiments, we used a dual-luciferase reporter assay to detect binding between *TTF1* and the *Kiss1* gene promoter, not the Kiss1 receptor (*Kiss1R*). *TTF1* bound to the *Kiss1* promoter and activated the transcription of the *Kiss1* gene. Interestingly, the expression of *GnRH* mRNA decreased significantly after the overexpression of *TTF1* in GT1–7 cells. Provenzano et al. [26] found that *TTF1* interfered with GnRH secretion by directly regulating Secreted Protein Acidic and Rich in Cysteine (*Sparc*) by binding to its promoter not directly through *Kiss1* in GT1–7 cells. There are other regulatory pathways linking *TTF1* and *GnRH*. This may explain why the expression of *GnRH* decreased after the upregulation of *TTF1* in GT1–7 cells. The relationship between TTF1 and GnRH can be explored in future studies.

Previous studies by our group found that the mRNA expression of *GnRH* in the ARC and AVPV continued to rise with puberty and reached a peak in the adult stage (PND42). However, the mRNA expression of *Kiss1* in the ARC and AVPV reached its peak in the early puberty stage (PND35) and remained at a high level at PND42. Furthermore, after the peak expression during juvenile development, TTF1 expression decreased at the early puberty stage, and its expression increased again in the adult hypothalamus [27]. To observe the regulatory role of *TTF1* in animals, we used stereotactic injection to deliver lentivirus bearing TTF1-shRNA into the ARC or AVPV nucleus of female rats at PND21. Stereotactic injection can deliver lentivirus into mammals to induce stable and continuous expression of the target fragment at specific locations. Knockdown but not complete ablation of *TTF1* expression in the ARC or AVPV nucleus preserved some physiological function of *TTF1*, and the potentially confounding effects of developmental compensation or redundancy were limited. In vivo experiments confirmed that we successfully injected LV-TTF1-shRNA into the ARC or AVPV nucleus of 21-day-old female rats through bilateral microinjections. Female rats that were injected with LV-TTF1-shRNA in the ARC or AVPV nucleus showed a pronounced reduction in *Kiss1* and *GnRH* mRNA abundance. However, there were no significant changes in Kiss1 protein among the TTF1-KD group at the juvenile stage. This may be related to the low expression of Kiss1 at this stage.

Vaginal opening signifies the rising oestrogen levels necessary for puberty initiation. *TTF1* gene knockdown in the ARC or AVPV nucleus delayed the day of vaginal opening. Previously, researchers found that female rats begin to enter puberty at PND32. However, in our study, the rats underwent vaginal opening earlier than in a previous study [28]. Vaginal opening began at nearly PND28 in the NC group, while vaginal opening began at nearly PND30 in the TTF1-KD group. However, we speculate that this may be related to the orthotopic injection of the nuclei performed when the rat was 21 days old. After all, this is a traumatic operation, and it may have caused vaginal opening to occur earlier than expected in all of the rats. However, there was no significant difference in the uterine organ coefficient, ovarian organ coefficient or AGD between the *TTF1* knockdown and control groups. Interestingly, the rats with *TTF1* knockdown in the AVPV nucleus showed decreased body weight in prepuberty compared to the control rats. The hypothalamus is the portion of the brain that serves as the centre of food intake regulation [29]. Previously, researchers found that TTF1 is mainly expressed in the ARC, ventromedial hypothalamic nucleus (VMH) and other nuclei of the hypothalamus postnatally and is closely related to feeding behaviour (food intake) [30]. Kim et al. [27] downregulated the expression of TTF1 and found a decrease in animal food intake and body weight. In 2011, they further discovered that TTF1 affects feeding behaviour via the melanocortin pathway [31]. The ARC and AVPV nuclei have opposing regulatory effects on feeding behaviour [32, 33]. Moreover, these results also provide convincing support for the present results. The rats in the AVPV-specific TTF1-KD group had a tendency towards weight loss, while the weight of the rats in the ARC-specific TTF1-KD group was not significantly different from that in the control group. We hypothesize that the downregulation of TTF1 in the AVPV leads to reduced feeding behaviour and weight loss in rats. This represents a critical neural system underlying the control of body weight and other functions [34]. In summary, TTF1 has a novel role in the regulation of feeding behaviour in the rat hypothalamus.

The formation of the corpus luteum (CL) in the ovary signifies that mammals have reproductive functions and have begun puberty. Our results suggest that pubertal development was delayed in TTF1-KD rats. Downregulation of *TTF1* in the AVPV appeared to have a greater effect than downregulation of *TTF1* in the ARC, whereas both TTF1-KD groups had delayed puberty and abnormal ovary development. Moreover, Mastronardi et al. [35] used Cre-loxP technology to knock out the *TTF1* gene in the hypothalamus and found that mice showed delayed puberty, reduced reproductive capacity, and a short reproductive span. The present study enriches our understanding of puberty onset and provides a novel theoretical basis for the treatment of precocious puberty in children.

## Conclusions

In vitro, *TTF1* directly regulated *Kiss1* expression in GT1–7 cells. In vivo, downregulation of *TTF1* in ARC and AVPV female rats not only reduced *Kiss1* and *GnRH* expression but also resulted in delayed vaginal opening and ovarian abnormalities. In summary, we provide experimental evidence that *TTF1* could control mammalian puberty onset.

## Data Availability

All data generated or analysed during this study are included in this published article. The datasets used and/or analysed during the current study are available from the corresponding author upon reasonable request.

## Abbreviations

ARC: Arcuate
AVPV: Anterior ventral paraventricular
TTF1: Thyroid-specific transcription factor-1
T/EBP: Thyroid-specific enhancer-binding protein
GnRH: Gonadotropin-releasing hormone
KD: Knockdown
OE: Overexpression
VO: Vaginal opening
HPG: Hypothalamic-pituitary-gonadal
GPR54: G protein-coupled receptor 54
LH: Luteinizing hormone
ERα: Oestrogen receptor α
KERKO: Kisspeptin-specific ERα knockout
3 V: Third ventricle
VMPO: ventromedial preoptic nucleus
LV-EGFP: Lentivirus-enhanced green fluorescent protein
PND: Postnatal day
DMEM: Dulbecco’s Modified Eagle’s Medium
FBS: Foetal bovine serum
LV-NC: Lentivirus- negative control
AGD: Anogenital distance
SDS-PAGE: Sodium dodecyl sulfate-polyacrylamide gel electrophoresis
PVDF: Polyvinylidene fluoride
TBST: Tris-buffered saline with 0.1%Tween 20
H&E: Haematoxylin-eosin
EGFP: Enhanced green fluorescent protein
TTF1-KD: TTF1-knockdown group
TTF1-OE: TTF1-overexpression group
NC: Negative control group
CL: Corpus luteum
Ac: Anterior commissure
ME: Medial eminence
Sparc: Secreted Protein Acidic and Rich in Cysteine
